# Organoid-guided evidence that umbilical cord MSC-derived extracellular vesicles restore alveolar repair in cigarette smoke-induced lung injury

**DOI:** 10.3389/fcell.2026.1710021

**Published:** 2026-03-17

**Authors:** Syahidatulamali Che Shaffi, Anan A. Ishtiah, Azim Patar, Badrul Hisham Yahaya

**Affiliations:** 1 Lung Stem Cell and Gene Therapy Group, Department of Biomedical Sciences, Pusat Kanser Tun Abdullah Ahmad Badawi (PKTAAB), Universiti Sains Malaysia, Penang, Malaysia; 2 Department of Neurosciences, School of Medical Sciences, Universiti Sains Malaysia, Kubang Kerian, Kelantan, Malaysia

**Keywords:** alveolar regeneration, cigarette smoke (CS), extracellular vesicles, lung organoids, umbilical cord mesenchymal stem cells

## Abstract

Chronic cigarette smoke (CS) disrupts epithelial homeostasis, fuels persistent inflammation, and impairs alveolar repair—hallmarks of COPD with few disease-modifying options. Extracellular vesicles (EVs) from human umbilical cord mesenchymal stem cells (hUC-MSCs) are emerging as cell-free modulators of regeneration, yet their impact on the CS-injured alveolus and alveolar type-2 (AT2) stem/progenitor programs remains unclear. We used a preclinical model of chronic CS exposure coupled with organoid-guided analyses to test whether hUC-MSC-derived EVs can restore epithelial regeneration while tempering injury-associated inflammation and remodeling. Following CS injury, animals received vehicle, hUC-MSCs, or purified hUC-MSC EVs; lungs were evaluated histologically (airway/parenchymal inflammation, emphysema-like change), by Masson’s trichrome (collagen deposition), and functionally using *ex vivo* epithelial organoids (organoid number/size, architecture, and AT2/AT1 marker balance). Transcriptomic profiling of organoid-derived RNA mapped pathway-level changes. CS induced robust immune-cell infiltration, increased collagen, and abnormal organoid phenotypes consistent with dysregulated progenitor activity. Post-injury EV treatment reduced inflammatory infiltrates and collagen, normalized organoid number and size, and restored AT2/AT1 lineage balance toward naïve patterns. At the molecular level, EVs dampened injury-upregulated circuits (including IL-17, PI3K–AKT–mTOR, MAPK, oxidative-stress and matrix-remodeling signatures) and enriched pathways associated with epithelial homeostasis and barrier integrity. Together, these data position hUC-MSC EVs as precision modulators of the injured alveolar niche that rebalance inflammation and re-engage endogenous regenerative programs. The organoid-guided, multi-scale readouts provide mechanistic insight and a translational rationale for EV-based regenerative therapeutics in smoke-induced lung injury and, by extension, COPD.

## Graphical Abstract

**Figure:**
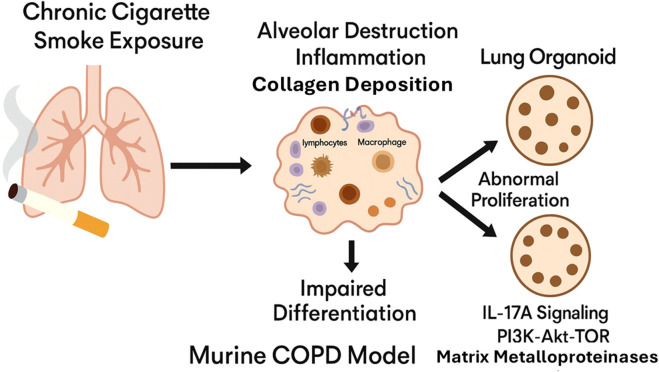
Integrative model summarizing the effects of chronic cigarette smoke exposure on lung inflammation, alveolar injury, dysregulated alveolar stem cell regeneration, and possible malignant transition toward lung cancer development.

## Introduction

Chronic obstructive pulmonary disease (COPD) remains a leading cause of global mortality and morbidity, driven largely by long-term exposure to cigarette smoke (CS), which induces persistent lung inflammation, oxidative stress, extracellular matrix degradation, and irreversible alveolar destruction culminating in emphysema (Halpin et al., 2019; Iheanacho et al., 2020; Su et al., 2022). Central to the maintenance of alveolar homeostasis are alveolar type 2 (AT2) epithelial cells, which function as facultative stem/progenitor cells capable of self-renewal and differentiation into alveolar type 1 (AT1) cells, the thin epithelial layer responsible for efficient gas exchange (Basil et al., 2020; Sasaki et al., 2018). Under physiological conditions, AT2-to-AT1 differentiation enables effective epithelial repair following acute injury. However, chronic injurious stimuli such as sustained CS exposure may impair AT2 regenerative capacity, disrupt epithelial–mesenchymal interactions, and contribute to progressive emphysema development and lung function decline (Mercado et al., 2015).

Despite extensive knowledge of COPD pathogenesis at the inflammatory and structural levels, the impact of prolonged CS exposure on lung progenitor cell behaviour at the cellular and molecular levels remains incompletely understood. In particular, the balance between effective regenerative repair and persistent tissue damage in COPD is poorly defined. Emerging evidence suggests that COPD is not solely a consequence of inflammatory destruction, but also reflects dysregulated epithelial regeneration and progenitor cell dysfunction, which may exacerbate disease progression.

Recent advances in three-dimensional (3D) lung organoid culture systems have provided powerful platforms for modelling alveolar regeneration *in vitro* (Kim et al., 2019; Shi et al., 2020; Kim et al., 2022). By recapitulating key aspects of lung architecture, cellular heterogeneity, and lineage differentiation, AT2-derived organoids allow mechanistic dissection of stem cell responses to chronic lung injury that cannot be achieved using conventional two-dimensional cultures. These systems have proven particularly valuable for interrogating progenitor activation, differentiation dynamics, and maladaptive repair processes associated with chronic lung diseases, offering novel insights into regenerative failure in COPD.

In this study, we utilized a murine model of CS-induced COPD combined with a 3D organoid culture system derived from lung epithelial progenitors to investigate the regenerative landscape under chronic smoke exposure. AT2 epithelial cells, known for their surfactant-producing capacity and progenitor-like regenerative potential, are particularly vulnerable to injury in chronic lung diseases. Previous studies have demonstrated that 3D organotypic coculture systems preserve AT2 cell structure and function more effectively than conventional 2D cultures, maintaining proliferative capacity, surfactant expression, and epithelial–mesenchymal interactions critical for lung repair (Sucre et al., 2018). Consistent with this, we have previously established a direct airway organoid culture platform suitable for modelling lung injury and regeneration in a preclinical setting (Shaffi et al., 2023).

Building upon our previous findings demonstrating that both human umbilical cord–derived mesenchymal stem cells (hUC-MSCs) and their extracellular vesicles (EVs) attenuate inflammation and structural lung damage in a rat model of COPD (Ridzuan et al., 2021), and supported by extensive evidence of their immunomodulatory and pro-reparative functions (Fujita et al., 2018; Abbaszadeh et al., 2022; Guo et al., 2021), we sought to address a more fundamental biological question: what is the fate and functional status of endogenous lung stem cells—particularly AT2 cells—under chronic cigarette smoke exposure? Under physiological conditions, AT2 cells serve as resident progenitors essential for alveolar maintenance and repair. However, persistent inhalation of cigarette smoke, as observed in chronic smokers who develop COPD, may compromise this regenerative capacity. We hypothesize that prolonged CS exposure either (i) impairs the self-renewal and differentiation capacity of resident lung stem/progenitor cells, or (ii) induces chronic overactivation that ultimately leads to progenitor exhaustion or maladaptive repair, thereby contributing to progressive alveolar destruction and disease pathogenesis.

To explore this hypothesis, we employed a murine model of cigarette smoke-induced COPD integrated with a 3D organoid culture system derived from lung epithelial progenitor cells. This organoid model provides a reductionist yet physiologically relevant platform to assess the functional competence of lung stem cells in response to injury and therapeutic intervention. We further evaluated the restorative potential of hUC-MSCs and their EVs—already proven to exert anti-inflammatory and immunomodulatory effects—on epithelial regeneration and alveolar repair. In parallel, transcriptomic profiling of organoid-derived RNA was performed to delineate key molecular pathways dysregulated in the context of impaired lung regeneration and modulated by therapeutic intervention.

Through this integrated *in vivo* and organoid-guided approach, our study aims to unravel how chronic smoke exposure disrupts lung stem cell function and regenerative capacity, providing deeper mechanistic insights into COPD progression and highlighting potential cellular and molecular targets for regenerative therapy.

## Methods

### Experimental model details

#### Animals

A total of 25 adult male BALB/c mice (8–12 weeks old, weighing 19–25 g) were used to develop the *in vivo* model of COPD. Mice were obtained and housed at the Animal Research Facilities (ARF), Advanced Medical and Dental Institute (AMDI), Universiti Sains Malaysia (USM) with proper lighting controlled on a 12 h light and 12 h dark cycle. The mice had access to food and water *ad libitum*. All animal experiments were granted ethical approval by the Institutional Animal Care and Use Committee (IACUC) of the Universiti Sains Malaysia [application number: USM/IACUC/2020/(122) (1050)].

### Cigarette smoke-induced COPD mouse model and treatment design

**FIGURE 1:**
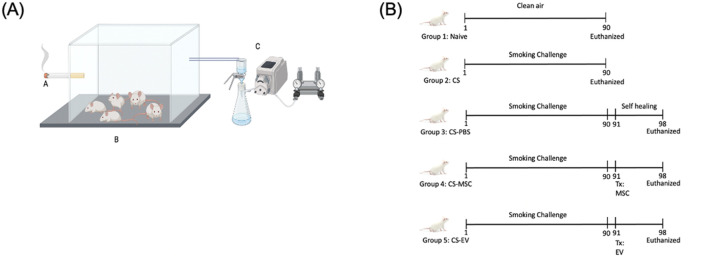
Experimental design and treatment timeline for the murine COPD model. **(A)** Smoking chamber design, **(B)** The experimental and treatment timeline for COPD model. Mice were divided into five groups: (1) naïve (clean air, no exposure), (2) CS (exposed to cigarette smoke for 12 weeks), (3) CS-PBS (exposed to cigarette smoke for 12 weeks followed by a 1-week self-healing phase with PBS injection), (4) CS-MSC (cigarette smoke exposure for 12 weeks followed by a single dose of hUC-MSCs, and (5) CS-EV (cigarette smoke exposure for 12 weeks followed by a single dose of hUC-MSC-derived EVs. All interventions (PBS, MSCs, EVs) were administered on day 90, followed by an observation period of 1 week. The naïve and CS groups were euthanized at day 90, while the CS-PBS, CS-MSC, and CS-EV groups were euthanized at day 98 to evaluate the effect of treatments.

To establish the murine COPD model, male BALB/c mice were exposed to commercially available Marlboro cigarettes (Philip Morris, United States), each containing 10.0 mg of tar and 1.0 mg of nicotine (Figure 1). Cigarette smoke (CS) exposure was conducted using a whole-body inhalation chamber, with mice exposed to the smoke of 10 cigarettes per session, twice daily (total of 20 cigarettes/day), 5 days per week for 12 consecutive weeks.

A total of five experimental groups (n = 5 per group) were assigned as follows.

1. Naïve group (unexposed control): maintained under clean air conditions and euthanized on Day 90.
2. CS group (injury model): subjected to 12 week CS exposure and euthanized on Day 90 without any intervention.
3. CS-PBS group (self-healing control): received PBS injection on Day 90 after completing smoke exposure and was euthanized on Day 98.
4. CS-MSC group: treated with 2 × 10^5^ human umbilical cord-derived mesenchymal stem cells (hUC-MSCs) via injection on Day 90 and euthanized on Day 98.
5. CS-EV group: treated with extracellular vesicles (EVs) isolated from 2 × 10^5^ hUC-MSCs, administered on Day 90, and euthanized on Day 98.

This design allowed evaluation of disease phenotypes at the injury peak (Day 90) and assessment of spontaneous *versus* treatment-induced recovery effects over an 8 day post-treatment period.

### Culture of hUC-MSCs and isolation of hUC-MSC-EVs

Human UC-MSCs at passage 5 were used for this study. Mesenchymal stem cells (2 × 105) were cultured in complete medium containing DMEM/F12 (Thermo Fisher Scientific, Massachusetts, United States) supplemented with 10% FBS (Thermo Fisher Scientific, Massachusetts, United States), 1% antibiotic antimycotic (Thermo Fisher Scientific, Massachusetts, United States), 1% L-glutamine (Thermo Fisher Scientific, Massachusetts, United States), and 20 ng/mL basic fibroblast growth factor (bFGF) (Thermo Fisher Scientific, Massachusetts, United States) and incubated in 5% CO_2_ at 37 °C. After 2 days, the media was changed to FBSEV-deprived complete media for the collection of the hUC-MSC-EVs. Before the medium was changed to FBSEV-free complete medium, the medium was prepared by centrifuging the cells in DMEM/F12 (Thermo Fisher Scientific, Massachusetts, United States) supplemented with 10% FBS (Thermo Fisher Scientific, Massachusetts, United States) at 100,000 × g for 18 h. The medium was collected in a 50 mL Corning™ Falcon tube (Thermo Fisher Scientific, Massachusetts, United States), and the pellet was discarded. After 2 days of culture, the FBEV-free medium was incubated at 4 °C until use. After 72 h, the MSC-CM was collected and subjected to differential centrifugation. Differential centrifugation of hUC-MSC-CM was conducted by using a Kubota 2420 Compact Tabletop Centrifuge (Kubota, Tokyo, Japan) at 300 × g for 10 min to remove dead cells. The collected supernatants were centrifuged again by using an Allegra X-15R Centrifuge Ultracentrifuge (Beckman Coulter, Indianapolis, United States) at 10,000 × g for 30 min to remove debris, followed by ultracentrifugation at 100,000 × g for 2 h to precipitate the hUC-MSC-EVs by using a Type 50.2Ti fixed-angle rotor and an Optima L-100K Ultracentrifuge (Beckman Coulter, Indianapolis, United States). The supernatants were discarded, and the hUC-MSC-EVs were washed with 1× PBS by ultracentrifugation at 100,000 × g for 1 h hUC-MSC-EVs were collected, resuspended in 150 μL of 1× PBS and freshly used for treatment. Characterization of hUC-MSC-EVs was conducted as previously described by our group (Ridzuan et al., 2021). EV morphology was assessed using EFTEM, CD63 expression was verified by western blotting, and particle size distribution was determined using nanoparticle tracking analysis (Nanosight NS300), with a mean vesicle diameter of approximately 153 nm.

### Collection of lung tissue

The mice were humanely euthanized, and immediately thereafter, their lungs were dissected using sterile equipment for sample collection. The lung collection process involved two separate steps: 1) The left lung was preserved for tissue histology analysis, while 2) the right lung was reserved for the development of an organoid culture. For tissue histology, the left lung was promptly immersed in 10% buffered formalin. Meanwhile, half of the right lung was carefully placed in a 15 ml tube containing ice-cold sterile phosphate-buffered saline (PBS). This PBS solution was supplemented with an antibiotic/antimycotic solution comprising 100 l/ml penicillin, 10 mg/ml streptomycin, and 25 g/ml amphotericin B from Sigma‒Aldrich, Missouri, United States, until further processing. This prepared the lung tissue for subsequent 3D alveolar organoid culture.

### Development of 3D alveolar organoid culture

The lung organoid culture was established based on previously published protocols developed by our group (Shaffi et al., 2023). Lung tissue collection was carried out by placing the lung in cold-ice sterile PBS and promptly transferring it to the cell culture room under sterile conditions. The lung tissue was minced until it reached a slurry-like consistency and then digested overnight at 4 °C with continuous shaking at 200 rpm in digestion medium. The digestion medium consisted of 1 mg/ml pronase (Gibco) and 100 μl/ml antibiotic/antimycotic solutions prepared in modified Hank’s balanced salt solution (HBSS) from Sigma‒Aldrich.

On the following day, the lung tissue epithelium was dislodged using a cell scraper and passed through a 70 μm cell strainer before being transferred to a 15 ml tube containing 0.25% trypsin/EDTA (Gibco). The lung tissue was then incubated for 5 min at 37 °C. To halt the digestion process, 10% FBS was added, and the cells were pelleted by centrifugation at approximately 600 × g for 5 min at 4 °C using an Eppendorf 5804 R centrifuge from Hamburg, Germany.

After resuspension, red blood cells were lysed using ammonium chloride (Sigma‒Aldrich) for 10 min, followed by centrifugation at approximately 600 × g for 5 min at 4 °C to remove the lysis buffer. The epithelial cell pellet was then washed twice with PBS and centrifuged again under the same conditions before being resuspended in complete culture medium for cell counting. A total of 50 × 10^3^ cells were by culturing the cells in Growth factor-reduced Matrigel™ (Corning, NY, United States). Two 15μL drops of the Matrigel-cell suspension were pipetted into wells of a Nunc 24-well Nuclon Delta-treated plate (Thermo Fisher Scientific) and allowed to solidify for 15 minutes at 37 °C. Then, 400 μl of lung organoid proliferation medium was added to the respective well. The cultures were monitored daily to assess the formation of organoids. The cells were grown for 6 days with medium change every three days. After day 6, the cell will form a spheroid. To promote the maturation and differentiation of the spheroids into organoids, the medium was changed to a medium containing 2% FBS (instead of 5% in the proliferation medium), with/without ROCK and TGF-β inhibitors.

The alveolar organoid-cell block was created by mixing the alveolar organoids with an in-house prepared CytoQuick Gel Kit solution, which was then centrifuged at 600 × g for 10 minutes. After a 10-minute solidification period at 4 °C, the resulting cell block was processed using an automated tissue histology processor. Tissues were embedded in paraffin and sectioned into 3 μm thick slices with a semiautomated rotary microtome (Leica Biosystems) for histological staining and immunostaining as required.

### Hematoxylin and eosin staining

For the investigation and scoring of peribronchial and perivascular inflammation, alveolar inflammation, and emphysema in the lung area, hematoxylin and eosin (H&E) staining was used. A standard H&E staining procedure was used to evaluate the structure and composition of the tissue. Peribronchial and perivascular lung inflammation were measured by a scoring technique. The score was determined based on the following parameters: 0, no inflammation detected; 1, occasional cuffing with inflammatory cells; 2, the lung was surrounded by a thin layer of inflammatory cells (1-5 cells thick); and 3, the lung was surrounded by a thick layer of inflammatory cells (>5 cells thick). Scoring of lung inflammation was conducted and guided by a histopathologist using semiquantitative analysis. The slides were blindly coded before inflammation scoring was performed. Parenchyma inflammation was measured by the griding method, and the score was calculated according to the following parameters: 0, when a normal number of cells was observed; 1, when a minimal amount of immune cells was observed; 2, when a mild amount of immune cells was observed; 3, when a moderate amount of immune cells was observed; and 4, when a severe amount of immune cells was observed. The scoring of lung inflammation was performed according to a previous study (Ridzuan et al., 2021). For lung emphysema, the area of alveolar destruction was measured by gridding using ImageJ according to a method described in a previous study (Ridzuan et al., 2021).

### Masson trichrome staining

To investigate collagen deposition in the lung tissue, Masson trichrome staining was performed. A standard protocol (Abcam) was followed according to the manufacturer’s instructions. The staining interpretation was as follows: blue staining indicated positive collagen staining, muscle fibers were stained red, and nuclei were stained dark red to black/blue. Ten images were captured for each group, and collagen deposition was quantified using ImageJ. To measure the collagen deposition area, a specific blue-stained area was chosen accordingly. The total area of the slide was measured before selecting the specific area of the blue staining and were determined by the color threshold method using ImageJ.

### Immunofluorescence staining

Sections of organoids were placed onto glass slides coated with poly-L-lysine. These sections were then heated to 60 °C to remove paraffin. Subsequently, the slides were immersed in xylene for 3 min to further eliminate paraffin. To rehydrate the samples, a series of ethanol solutions (100%, 90%, 80%, and 70%) and PBS were used, each for 3 min.

Following paraffin removal and rehydration, the slides were subjected to antigen retrieval by being placed in 10 mM citrate buffer. The samples were incubated at 95 °C for 20 min to expose the antigenic sites. After cooling, the slides were washed with PBS for 5 min. To enhance permeability, the slides were treated with 0.25% Triton X-100 (Sigma‒Aldrich) in PBS for 10 min at room temperature. Subsequently, a 5% bovine serum albumin (BSA; Sigma‒Aldrich) solution in PBS-Tween 20 (Sigma‒Aldrich) was applied to block nonspecific binding for 20 min.

For antibody labeling, primary antibodies specific for Sftpc (1 mg/ml; Elabscience) and Aqp5 (200 μg/ml; Santa Cruz) were used for lung organoids. These primary antibodies were diluted in PBS-Tween 20 containing 1% BSA, added to the slides, and incubated overnight at 4 °C in a humidified chamber. After incubation, the slides were washed twice with PBS for 5 min each. Subsequently, appropriate secondary antibodies, which were also diluted in PBS supplemented with 1% BSA, were applied to the slides. Following an additional 40 min incubation at room temperature, the slides were washed twice with PBS. To visualize the cell nuclei, the samples were counterstained with 4′,6-diamidino-2-phenylindole (DAPI) (Sigma‒Aldrich) for 5 min at room temperature. Finally, the slides were mounted using Fluoroshield™ (Sigma‒Aldrich). The stained samples were observed using a bright-field phase-contrast/fluorescence inverted TI-U microscope (Nikon, Tokyo, Japan). Image analysis and processing were carried out using NIS-Element software (Nikon).

### RNA extraction and microarray analysis

RNA was extracted from three different groups: naïve day 14 (ND14), CS day 5 (CSD5), and CS day 14 (CSD14). This was accomplished using the NucleoSpin RNA Plus Extraction Kit (Macherey Nagel, Germany) following the provided guidelines. To assess RNA purity and concentration, a NanoDrop ND100 instrument (Thermo Fisher Scientific, United States) was utilized. Subsequently, 10 ng of the obtained total RNA was subjected to reverse transcription to generate first-strand cDNA. First-strand cDNA was used to create double-stranded cDNA via *in vitro* transcription (IVT). This cDNA underwent IVT amplification to generate cRNA, which was purified for 2nd-cycle single-stranded cDNA synthesis. The fragmented, labeled cDNA was hybridized to the Mouse Gene 2.0 ST Array for 18 h at 45 °C. The arrays were subsequently scanned using an Applied BiosystemsTM GeneChipTM Scanner 3000 7G.

For microarray data analysis, RNA isolation and processing were performed using Transcriptome Analysis Console (TAC) software version 4.0.3 (Thermo Fisher Scientific, Massachusetts, United States). Significantly altered sample datasets were determined using a significance threshold of p < 0.05. Genes exhibiting both p < 0.05 and a fold change greater than 2.0 were considered significantly differentially expressed. Various graphical representations, such as volcano plots, heatmaps, Venn diagrams, and pathway analyses, were generated using TAC software to visualize the results of the analysis.

### Statistical analysis

All quantitative data were analysed using GraphPad Prism version 9.0 (GraphPad Software, CA, United States). Experimental groups included: naïve (clean air control), cigarette smoke–exposed (CS), CS-PBS, CS-MSC, and CS-EV (n = 5 animals per group unless otherwise stated).

For comparisons involving more than two experimental groups of lung organoid, statistical significance was assessed using one-way analysis of variance (ANOVA). When ANOVA indicated a significant overall effect, Tukey’s multiple-comparison *post hoc* test was applied to determine pairwise differences between groups. Key planned comparisons included CS *versus* naïve (injury effect), CS *versus* CS-MSC or CS-EV (treatment effects).

Histological inflammation scores, inflammatory cell counts, emphysema indices, collagen deposition area, lung organoid number, and organoid size were all analysed using this approach. Data are presented as mean ± standard deviation (SD). Correlation analyses between organoid parameters and airway inflammation scores were performed using Pearson’s correlation coefficient.

For all analyses, a p value ≤0.05 was considered statistically significant. Levels of significance are indicated in the figures as p ≤ 0.05, p ≤ 0.01, p ≤ 0.001, and p ≤ 0.0001.

## Results

### hUC-MSCs and EVs significantly attenuate peribronchial and perivascular inflammation induced by chronic cigarette smoke exposure

**FIGURE 2:**
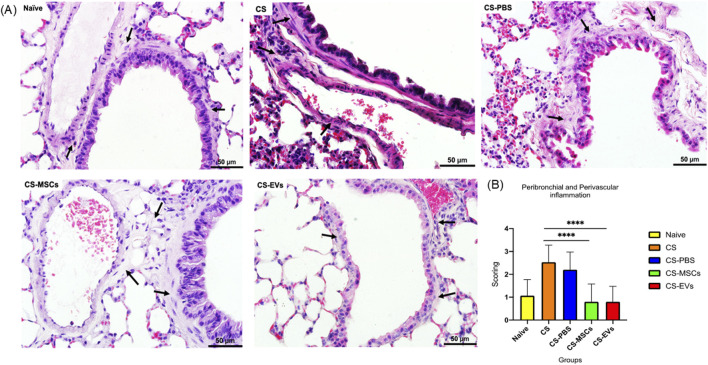
MSCs and EVs attenuate cigarette smoke-induced peribronchial and perivascular inflammation in lung tissue. **(A)** Representative images of hematoxylin and eosin (H&E) stained lung sections from the Naïve, CS (Cigarette Smoke), CS-PBS, CS-MSCs, and CS-EVs groups. Black arrows indicate regions of inflammatory cell infiltration around the airways and blood vessels. Increased wall thickening and leukocyte recruitment are observed in the CS and CS-PBS groups, which are noticeably reduced in groups treated with MSCs or EVs. Scale bars = 50 μm. **(B)** Histological scoring of peribronchial and perivascular inflammation across experimental groups. Data are presented as mean ± SD. Statistical significance was determined using a one-way ANOVA followed by Tukey’s test. ****P < 0.0001.

Characterization of hUC-MSC-derived EVs was performed as previously described by Ridzuan et al. (2021). Briefly, EVs exhibited a rounded morphology under EFTEM, expressed the exosomal marker CD63 as confirmed by western blotting, and showed a size distribution consistent with small extracellular vesicles, with an average diameter of approximately 153 nm as determined by nanoparticle tracking analysis. Chronic cigarette smoke exposure resulted in pronounced peribronchial and perivascular inflammation, as evidenced by thickened cellular infiltrates surrounding airways and blood vessels in H&E-stained lung sections–Figure 2. These pathological features were markedly increased in the CS and CS-PBS groups compared to the naïve controls, confirming the establishment of a COPD-like inflammatory phenotype. Semi-quantitative histological scoring revealed a significant elevation in inflammation scores in both groups (p < 0.0001 vs. naïve), reflecting persistent inflammatory responses even after cessation of CS exposure–Figure 2.

Intervention with hUC-MSCs or their EVs markedly suppressed this inflammation. Histological images from the CS-MSC and CS-EV groups displayed visibly reduced immune cell infiltration in the peribronchial and perivascular regions. Corresponding inflammation scores showed a statistically significant decrease in both treatment groups compared to the CS group (*p* < 0.0001), returning toward levels observed in the naïve group.

These results demonstrate that both hUC-MSCs and EVs are sufficient to reverse cigarette smoke-induced peribronchial and perivascular inflammation, thereby confirming their potent anti-inflammatory efficacy and disease-modifying potential in this murine COPD model.

### hUC-MSCs and EVs mitigate inflammatory cell infiltration and alveolar destruction in cigarette smoke-exposed lungs

**FIGURE 3:**
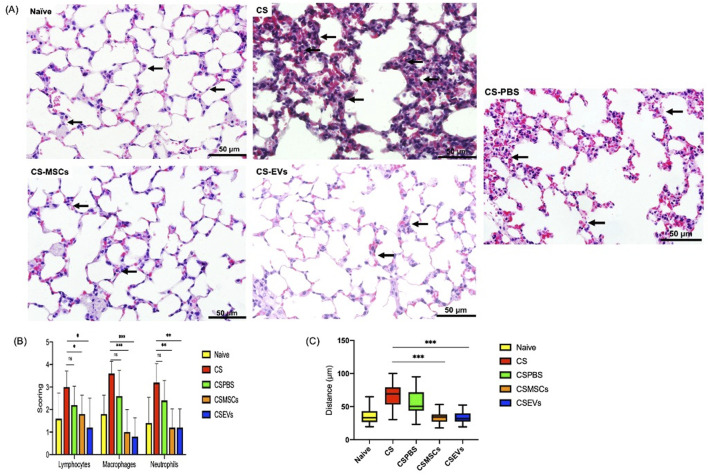
The effects of lung parenchymal inflammation and emphysematous changes following hUC-MSC and EV treatment. The number of lymphocytes, macrophages, neutrophils, and the area of emphysema are reduced after CS-MSCs and CS-EVs treated groups. The arrows showed the representative area of histological images of parenchyma area. Large, irregular alveolar spaces due to alveolar wall destruction, particularly in the CS and CS-PBS groups. The arrows also highlight enlarged airspaces, suggesting emphysematous change as a result of cigarette smoke exposure. **(A)**. Semi quantitative histology analysis of lung parenchymal inflammation including lymphocytes, macrophages, neutrophils and emphysema of the alveolar **(B)**. The infiltration of inflammatory cell of lung parenchymal area significantly reduced in treatment group (CS-MSCs and CS-EVs) (***p < 0.001) compared to the CS group. **(C)**. The significant reduction in alveolar destruction from treatment groups (****p < 0.0001). Data shown as absolute number as median ± SD, N=5 which consisted of 5 mice per each group.

Histological analysis of lung parenchyma revealed that CS exposure induced prominent pathological changes characteristic of emphysema, including destruction of alveolar walls and widespread infiltration of inflammatory cells such as lymphocytes, macrophages, and neutrophils (Figure 3). These effects were markedly attenuated in mice treated with hUC-MSCs or their EVs. Representative H&E-stained images demonstrated large, irregular airspaces and thickened alveolar septa in the CS and CS-PBS groups, indicative of alveolar destruction and inflammation (Figure 3). In contrast, the CS-MSC and CS-EV treatment groups exhibited relatively preserved alveolar architecture with reduced inflammatory infiltrates.

Quantitative analysis supported these observations: both hUC-MSC and EV-treated groups showed significant reductions in the number of lymphocytes, macrophages, and neutrophils compared to the untreated CS group (*p* < 0.001) ‐ Figure 3. Furthermore, the alveolar destruction index, used to assess the severity of emphysema, was significantly decreased following both interventions (*p* < 0.0001) – Figure 3C, indicating that the treatments were effective in preserving alveolar structure and mitigating disease progression. These results highlight the capacity of hUC-MSCs and EVs to modulate the inflammatory microenvironment and limit emphysematous damage induced by prolonged cigarette smoke exposure, thus demonstrating their therapeutic relevance in COPD-like conditions.

### hUC-MSC and EV treatments effectively attenuate cigarette smoke–induced collagen deposition in lung tissue

**FIGURE 4:**
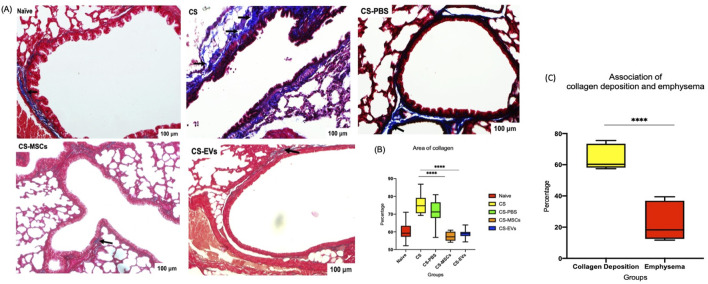
hUC-MSC and EV treatments reduce collagen deposition induced by cigarette smoke exposure. **(A)** Cigarette smoke group shows an increase in collagen deposition. The arrows showed the representative of microscopic analysis of Masson trichrome staining of naïve, CS, CS-PBS, CS-MSCs group and CS-EVs group. **(B)** Quantification analysis of the area of collagen in each group and a substantial increase in collagen deposition was discerned in the CS group in comparison to the other groups with p < 0.0001. **(C)** There is an association between collagen deposition after CS and lung emphysema paradigm. Bar graph represented the association of collagen deposition and lung emphysema representing all groups including injury and treatment groups (****p < 0.0001).

Masson’s trichrome staining revealed marked increases in collagen deposition in the lungs of mice exposed to chronic CS, particularly in the CS and CS-PBS groups. Representative histological sections showed intensified blue staining around the airways and interstitial regions, indicative of fibrotic remodelling associated with chronic inflammation–Figure 4. Quantitative morphometric analysis confirmed that the collagen-positive area was significantly elevated in the CS group compared to the naïve control (*p* < 0.0001), validating the fibrogenic effect of prolonged CS exposure–Figure 4. Treatment with hUC-MSCs or their EVs led to a substantial reduction in collagen deposition. Both CS-MSC and CS-EV groups exhibited a clear decrease in collagen area percentage compared to the CS group (*p* < 0.0001), approaching levels observed in naïve lungs. These improvements suggest that the interventions are sufficient to reverse fibrotic remodelling triggered by cigarette smoke.

Collectively, these findings demonstrate that both hUC-MSCs and EVs possess strong anti-fibrotic effects *in vivo*, capable of mitigating the structural remodelling of lung tissue—a hallmark of COPD pathogenesis–Figure 4. Their application not only alleviates inflammation but also interrupts downstream fibrotic processes that contribute to chronic lung injury.

### Cigarette smoke induces aberrant lung organoid formation, which is normalized by hUC-MSC and EV treatments

**FIGURE 5:**
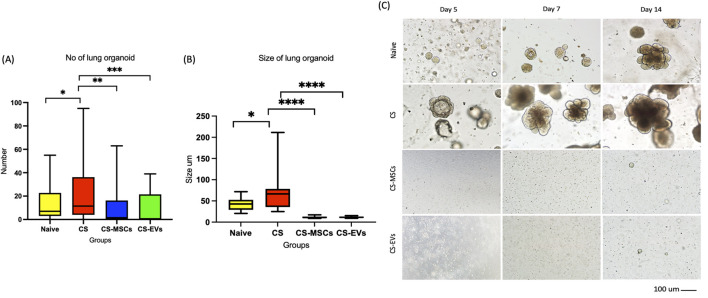
MSCs and EVs influence lung organoid formation and morphology following cigarette smoke exposure. **(A)** Total number of lung organoids formed per well across experimental groups. The CS group shows a significant increase in organoid initiation compared to the Naïve group, which is attenuated by MSC and EV treatment. **(B)** Average size of lung organoids measured in micrometers (μm). Box plots represent the median and interquartile range, with whiskers indicating the [insert e.g., Min to Max] range. Note the significant reduction in organoid size in the CS-MSCs and CS-EVs groups compared to the CS group. **(C)** Representative bright-field microscopy images of lung organoid cultures at Day 5, Day 7, and Day 14. Organoids in the Naïve and CS groups show progressive growth and complex morphology, while MSC and EV treated groups exhibit minimal organoid development over the 14-day period. Scale bar = 100 μm. Data are expressed as Mean ± SD. Statistical significance was analyzed using a one-way ANOVA followed by Tukey’s post-hoc test. *p < 0.05, **p < 0.01, ***P < 0.001, ****P < 0.0001.

To evaluate the impact of chronic CS exposure on lung epithelial regenerative potential, we employed a 3D organoid culture system using lung progenitor-enriched cells isolated from different treatment groups. Organoid formation efficiency and morphology varied markedly across experimental groups, reflecting changes in the proliferative and regenerative responses of alveolar progenitors–Figure 5.

Mice exposed to CS (CS group) exhibited a significantly higher number and larger size of organoid spheres compared to naïve controls (p < 0.001), suggesting hyperactivation of epithelial progenitor cells likely driven by sustained inflammation and injury. In contrast, lung cells from the hUC-MSC and EV treatment groups (CS-MSC and CS-EV) generated fewer and smaller organoids, with a significant reduction in both metrics compared to the CS group (p < 0.001), and approaching levels seen in naïve cultures–Figure 5.

**FIGURE 6:**
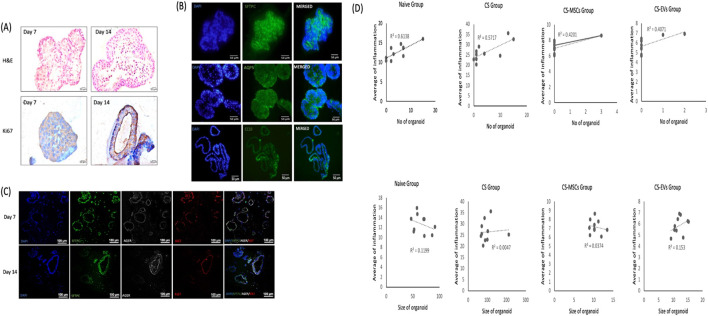
Representative histological and immunostaining analyses of lung organoids and their association with airway inflammation across experimental groups. **(A)** The images of H&E and IHC staining of Ki67 of organoid on day 7 and day 14 from CS group. **(B)** IF staining on Sftpc marker for AT2 cell, AQP5 marker for AT1 cell and CC10 marker for Club cell of the lung organoid from CS group. **(C)** IF staining on Sftpc, AGER, and Ki67 on lung organoid from naïve group. **(D)** The graph illustrates the relationship between organoid formation and airway inflammation status for various experimental groups. Significant correlations (p < 0.05 and p < 0.01) were observed in all groups, highlighting the association between organoid formation and inflammation. Conversely, when assessing the relationship between lung size and lung tissue inflammation, no significant correlation was evident across all groups (p > 0.05) (ns).

The reduced organoid formation following hUC-MSC or EV treatment is not indicative of impaired regenerative potential, but rather a normalization of the epithelial response in the context of resolved inflammation. This interpretation is supported by the strong positive correlation between the number of organoids and histological inflammation scores (Figure 6), where heightened inflammation in CS lungs coincided with increased organoid output. Conversely, the anti-inflammatory effects of hUC-MSCs and EVs, as shown in earlier figures, were associated with restored tissue homeostasis and less compensatory epithelial activation.

These findings suggest that while CS stimulates excessive stem/progenitor activation due to injury, hUC-MSC and EV treatments restore physiological regenerative dynamics by suppressing inflammation and rebalancing epithelial responses.

### Lung organoid histology and AT2 marker expression reflect inflammatory status and regenerative dynamics across treatment groups

To further assess the regenerative responses of lung progenitor cells following chronic CS exposure and subsequent treatment, we conducted histological and immunostaining analyses of organoids derived from each experimental group. H&E-stained sections revealed distinct morphological differences between groups–Figure 6. Organoids from the CS group exhibited a hyperplastic, multilayered epithelial architecture, consistent with heightened regenerative activity likely driven by persistent inflammation. In contrast, organoids derived from hUC-MSC- and EV-treated groups (CS-MSC and CS-EV) displayed more uniform, monolayered epithelial structures, resembling those of the naïve group, indicative of normalized regenerative responses following inflammation resolution.

Immunohistochemical staining for surfactant protein C (SP-C), a marker of AT2 cells, was performed to evaluate progenitor cell identity. SP-C expression was prominent in the CS group, consistent with increased AT2 activation–Figure 6, but was notably reduced in the CS-MSC and CS-EV groups, mirroring the histological trend of lower organoid number and size seen in Figure 5. This observation supports the hypothesis that hUC-MSC and EV treatments suppress aberrant AT2 cell overactivation by mitigating inflammation and restoring tissue homeostasis.

Moreover, correlation analysis (Figure 6) between organoid number and airway inflammation score demonstrated a strong positive association (*p* < 0.01), reinforcing the conclusion that regenerative output is directly influenced by the inflammatory milieu. These findings highlight the dual role of inflammation in driving epithelial regeneration and how effective anti-inflammatory interventions recalibrate this response to a more physiological state.

### Transcriptomic profiling of lung organoids reveals inflammation-associated molecular signatures modulated by hUC-MSC and EV treatments

**FIGURE 7:**
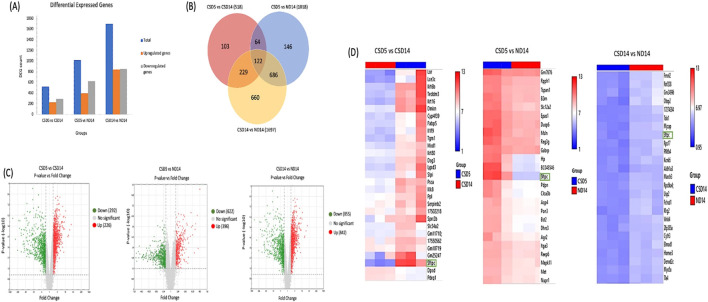
Comparative gene expression analysis of lung organoids reveals key regulatory genes involved in inflammation. **(A)** The differential expressed gene of comparison between CSD5 vs CSD14, CSD5 vs ND14 and CSD14 vs ND14. **(B)** Venn diagram shows overlapping DEG among CSD5 vs CSD14, CSD5 vs ND14 and CSD14 vs ND14. **(C)** Volcano plot of microarray data displaying the pattern of the gene expression profile in the lung organoid from three different groups which are CSD5 vs CSD14, CSD5 vs ND14 and CSD14 vs ND14. **(D)** Representative heatmap of key factors involved in regulation of the inflammatory response based on the above microarray data. Red, relatively up-regulated expression; blue, relatively down-regulated expression. Each column represents one individual sample, and each row represents one single gene. n = 3 per group.

To gain molecular insights into the regenerative responses of lung epithelial progenitors under chronic CS exposure and following stem cell-based therapy, we performed comparative gene expression profiling of lung organoids using microarray analysis. Hierarchical clustering and principal component analysis (PCA) revealed distinct gene expression patterns between the groups. Organoids derived from the CS group demonstrated a unique transcriptomic signature, clearly segregated from the naïve and treatment groups (CS-MSC and CS-EV), indicating that CS exposure induces a robust alteration in gene expression reflective of epithelial stress and inflammatory activation–Figure 7.

Functional enrichment analysis of the differentially expressed genes in the CS group revealed significant upregulation of inflammatory and immune-related pathways, including TNF signalling, NF-κB activation, and cytokine–cytokine receptor interactions. Notably, several key pro-inflammatory mediators—IL-1β, CXCL10, CCL2, and ICAM1—were markedly upregulated in CS-derived organoids, supporting the histological findings of heightened inflammation and regenerative hyperactivation.

In contrast, treatment with hUC-MSCs or their derived EVs led to partial or full normalization of these pathways. Gene expression in the CS-MSC and CS-EV groups showed significant downregulation of pro-inflammatory genes, along with upregulation of pathways associated with epithelial homeostasis and cellular repair, such as TGF-β signalling and tight junction assembly. These molecular signatures are consistent with the observed reduction in organoid number and size (Figure 5) and the restored epithelial architecture (Figure 6), further supporting the efficacy of these interventions. Together, the transcriptomic findings establish a mechanistic link between cigarette smoke-induced inflammatory injury and dysregulated epithelial regeneration. More importantly, they demonstrate that both hUC-MSC and EV treatments effectively suppress these maladaptive transcriptional programs, thereby restoring a regenerative balance in lung epithelial progenitors.

Pathway enrichment analysis highlighted significant activation of IL-17A signalling, PI3K-Akt-mTOR, MAPK, oxidative stress, matrix metalloproteinases, and ECM remodelling pathways (Table 1). Key upregulated genes included *Sftpc*, *Mmp9*, *Mmp12*, *Il1a*, *Akt3*, and *Pik3r1* (Table 2), reflecting concurrent pro-regenerative and pro-inflammatory signalling.

**TABLE 1:** The regulated pathways associated with CS induction in lung organoids.

Pathways	P value		
	CSD5 vs CSD14	CSD5 vs ND14	CSD14 vs ND14
Striated muscle contraction	1.0E-06	0.013113	—
Comprehensive IL-17A signaling	0.009609	0.057415	—
Lung fibrosis	0.000481	0.000863	0.047723
Spinal cord injury	0.005277	0.001409	0.000072
Microglia pathogen phagocytosis pathway	0.000422	0.000262	0.018437
Tyrobp causal network in microglia	0.002079	0.000012	0.009909
Burn wound healing	0.00066	0.000127	0.043595
Oxidative stress and redox pathway	0.055626	0.002439	1.0E-06
Matrix metalloproteinases	0.00101	0.001924	—
Cholesterol metabolism with bloch and kandutsch-russell pathways	0.0495	5.0E-06	1.0E-06
Hypertrophy model	0.003504	0.003142	—
Retinol metabolism	0.022611	2.4E-05	0.000139
Blood clotting cascade	0.038154	—	—
Eicosanoid synthesis	0.034689	0.020593	0.002374
Macrophage markers	0.010008	0.000175	0.012762
Protein-protein interactions in podocytes	—	1.0E-06	1.0E-06
Adar1 editing defficiency immune response	—	1.0E-05	1.0E-06
Toll-like receptor signaling pathway	—	1.0E-06	0.000069
Cholesterol biosynthesis	—	1.0E-06	1.0E-06
Sphingolipid metabolism (integrated pathway)	—	0.000001	0.000026
Sphingolipid metabolism overview	—	0.000001	0.000026
Protein-protein interactions in the podocyte	—	0.000025	1.0E-06
Focal adhesion	—	0.000218	0.010395
Chemokine signaling pathway	—	0.000296	—
Mapk signaling pathway	—	0.000306	0.000024
Mapk signaling pathway	—	0.000488	0.000069
Insulin signaling	—	0.000553	0.000252
Alpha 6 beta 4 integrin signaling pathway	—	0.001171	—
Elongation of (very) long chain fatty acids	—	0.001304	—
Matrix metalloproteinases	—	0.001924	—
Focal adhesion: PI3K-Akt-mTOR signaling pathway	—	0.001994	0.000594
T cell receptor signaling pathway	—	0.00325	0.02803
Circulating monocytes and cardiac macrophages in diastolic dysfunction	—	0.005675	0.015094
Na/K-ATPase/Src signaling	—	0.006497	0.024948
Oxidative stress response	—	0.01232	0.015774
Inflammatory response pathway	—	0.013869	—
Integrin-mediated cell adhesion	—	0.014361	0.041726
Factors and pathways affecting insulin-like growth factor (IGF1)-Akt signaling	—	0.017325	—
Immune response in Tg26 glomeruli	—	0.017748	—
EGFR1 signaling pathway	—	0.027325	—
Regulation of actin cytoskeleton	—	0.029243	0.039518
Fatty acid biosynthesis	—	0.030536	—
Eicosanoid lipid synthesis map	—	0.030672	0.009289
mRNA processing	—	0.033181	0.021861
Parkinson’s disease	—	0.033392	0.0501
Alzheimer’s disease	—	0.03418	—
GDNF/RET signaling axis	—	0.034317	—
Oxidation by cytochrome P450	—	0.036211	—
Adipogenesis genes	—	0.039003	0.000187
PPAR signaling pathway	—	0.044368	0.00993
Endochondral ossification	—	0.045155	0.000317
Metapathway biotransformation	—	0.048459	—
IL-7 signaling pathway	—	0.048815	—
Eicosanoid metabolism via lipoxygenases (LOX)	—	0.056612	0.055066
Type II interferon signaling (IFNG)	—	—	0.000006
Nuclear receptors in lipid metabolism and toxicity	—	—	0.003949
Transcriptional activation by Nfe2l2 in response to phytochemicals	—	—	0.004779
p38 Mapk signaling pathway	—	—	0.007496
Nuclear receptors	—	—	0.012907
Ovarian infertility	—	—	0.020726
IL-5 signaling pathway	—	—	0.026302
SREBF and miR33 in cholesterol and lipid homeostasis	—	—	0.027075
B cell receptor signaling pathway	—	—	0.027118
Glycogen metabolism	—	—	0.029888
Primary focal segmental glomerulosclerosis (FSGS)	—	—	0.03291
Eicosanoid metabolism via cyclooxygenases (COX)	—	—	0.033426
GPCRs, class a rhodopsin-like	—	—	0.035793
Dysregulated miRNA targeting in insulin/PI3K-AKT signaling	—	—	0.043595
G13 signaling pathway	—	—	0.045549
Oxidative damage response	—	—	0.059973

Fifteen pathways demonstrated substantial regulation when comparing CSD5 and CSD14. 53 pathways displayed significant regulation in response to CSD5 vs ND14, and 51 pathways exhibited significant regulation in response to CSD14 vs ND14. Pathway with p < 0.05 is considered as significantly regulated.

**TABLE 2:** Gene with highest frequency in group CSD5 vs CSD14, CSD5 vs ND14 and CSD14 vs ND14.

Group	Gene	P value	FC	Frequency
CSD5 vs CSD14	Il-1a	2.00E-04	7.15	7
	Mmp9	1.22E-02	2.24	4
	Acta1	4.70E-03	10.38	3
	mmp12	1.00E-03	69.59	3
	ccl3	2.27E-02	5.65	3
	Sftpc	5.21E-05	140.48	1
CSD5 vs ND14	Pik3r1	3.50E-03	2.31	13
	Akt3	1.60E-03	5.24	9
	Spp1	1.33E-02	3.07	9
	mmp9	6.10E-03	2.65	6
	Lamb2	1.22E-02	2.08	6
	lama5	3.80E-03	2.08	4
	Mapk11	5.50E-03	2.23	3
	ccl3	1.49E-02	6.21	3
	mmp12	2.90E-03	38	2
	Nr2f2	2.00E-03	3.24	2
	Sftpc	6.01E-07	48.38	1
CSD14 vs ND14	Pik3r1	3.50E-03	2.31	13
	Akt3	1.60E-03	5.24	9
	Spp1	1.33E-02	3.07	9
	mmp9	6.10E-03	2.65	6
	Lamb2	1.22E-02	2.08	6
	lama5	3.80E-03	2.08	4
	Mapk11	5.50E-03	2.23	3
	ccl3	1.49E-02	6.21	3
	mmp12	2.90E-03	38	2
	Nr2f2	2.00E-03	3.24	2
	Sftpc	6.01E-07	48.38	1

## Discussion

This study provides novel mechanistic insights into how chronic cigarette smoke exposure alters alveolar epithelial regeneration and promotes maladaptive remodelling in COPD. Using a murine model of sustained CS exposure, we demonstrated that prolonged injury activates persistent inflammatory responses while simultaneously inducing abnormal AT2 cell proliferation and regenerative attempts.

While prior studies have extensively characterized inflammatory pathways in COPD, few have examined the direct consequences of chronic injury on lung progenitor behaviour. Our 3D organoid model revealed that AT2 cells derived from CS-exposed lungs exhibited hyperproliferative organoid growth, consistent with a compensatory response to sustained injury. However, this regenerative response appears dysregulated, as reflected by abnormal growth patterns, emphasizing that persistent injury may drive both progenitor activation and exhaustion over time (Tsutsumi et al., 2020; Yokohori et al., 2004).

MSCs and EVs demonstrated potent immunomodulatory effects, reducing inflammatory infiltration and collagen deposition while normalizing organoid growth. The exogenous cells and EVs may provide potent paracrine cues that directly promote epithelial regeneration, suppress deleterious inflammation and these cues may re-educate resident pulmonary mesenchymal cells, enhancing their proliferative and regenerative capacity and coordinating a more effective epithelial repair program (Ridzuan et al., 2021; Fujita et al., 2018; Abbaszadeh et al., 2022). The regenerative potential of airway mesenchyme, it is plausible that endogenous mesenchymal remodeling plays a substantial role here, with exogenous hUC-MSC/EVs acting as modulators rather than sole drivers of repair. These findings support the therapeutic potential of cell-based interventions to modulate the inflammatory niche and restore balanced regeneration in COPD (Ridzuan et al., 2021; Fujita et al., 2018; Abbaszadeh et al., 2022; Guo et al., 2021; Liu et al., 2017; Yáñ et al., 2015).

Importantly, transcriptomic analysis revealed robust activation of IL-17A signalling—a critical pathway implicated in COPD progression via neutrophilic inflammation, MMP activation, and matrix degradation (Ritzmann et al., 2022; Liu et al., 2021). In parallel, PI3K-Akt-mTOR and MAPK pathways were highly dysregulated, linking chronic inflammation to abnormal cell proliferation, senescence, and ECM remodelling (Iyoda et al., 2010). Upregulation of *Mmp9* and *Mmp12* further supports ongoing proteolytic injury contributing to emphysema progression (Gharib et al., 2018).

Collectively, our findings suggest that chronic CS exposure creates a vicious cycle of unresolved inflammation, proteolytic matrix degradation, and dysregulated epithelial regeneration. The persistent activation of AT2 cells may contribute not only to emphysema but also to increased cancer susceptibility, given the well-established role of AT2 cells as potential cells-of-origin for lung adenocarcinoma (Yokohori et al., 2004; Parris et al., 2019; Wilson et al., 2008; Chubachi et al., 2017).

This integrative model combining *in vivo* injury, 3D organoid culture, and transcriptomic profiling provides a powerful platform to dissect impaired regeneration in COPD and may inform precision-targeted regenerative therapies aimed at restoring lung homeostasis.

Chronic cigarette smoke exposure disrupts alveolar regenerative capacity through sustained inflammation, aberrant AT2 cell proliferation, and dysregulated activation of key inflammatory and proteolytic pathways. Our integrative model system—combining long-term smoke exposure, 3D lung organoid culture, and transcriptomic profiling—provides a robust experimental platform to dissect the impaired regenerative landscape in COPD.

**FIGURE 8:**
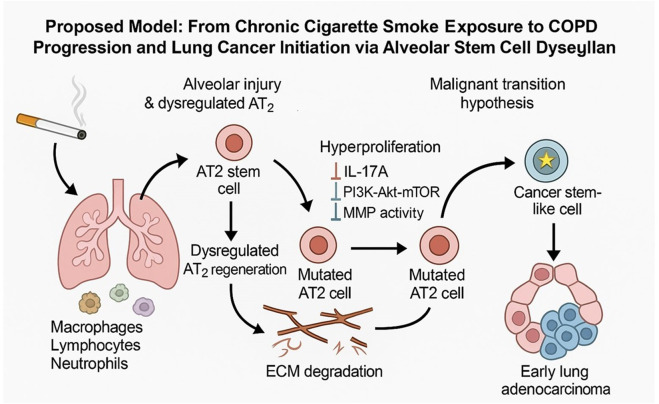
Conceptual model illustrating the proposed transition from chronic cigarette smoke exposure to COPD progression and lung cancer initiation via alveolar stem cell dysregulation.

Importantly, this model also offers an opportunity to explore a critical and emerging hypothesis: that chronic smoke-induced injury not only exhausts lung progenitor cells but may also reprogram surviving AT2 cells toward malignant transformation. The persistent hyperproliferative state, coupled with ongoing DNA damage, oxidative stress, and proteolytic remodeling, creates a permissive microenvironment for accumulating somatic mutations in lung stem cells. Over time, such genetic and epigenetic insults may drive a subset of AT2 cells to acquire cancer stem cell-like properties, seeding early lesions of lung adenocarcinoma—a well-documented clinical association in smokers with COPD–Figure 8.

By leveraging this model system, future studies can manipulate specific genetic pathways and inflammatory signals to directly assess how progenitor exhaustion, chronic inflammation, and oncogenic reprogramming converge to drive the transition from COPD to lung cancer. Ultimately, insights gained may inform precision prevention strategies aimed at intercepting malignant transformation in high-risk COPD populations.

Chronic cigarette smoke exposure leads to persistent lung inflammation characterized by infiltration of macrophages, lymphocytes, and neutrophils, as well as oxidative stress and DNA damage in alveolar epithelial cells. Alveolar type 2 (AT2) stem cells, responsible for maintaining alveolar integrity, enter a hyperproliferative state in response to sustained injury, driven by dysregulation of key signaling pathways including IL-17A, PI3K-Akt-mTOR, and matrix metalloproteinases (MMPs). Over time, accumulated genetic and epigenetic mutations may convert a subset of AT2 cells into mutated progenitors with impaired regenerative function. This dysregulated regenerative environment, coupled with extracellular matrix (ECM) degradation, may promote malignant transformation of mutated AT2 cells into cancer stem-like cells, initiating early lung adenocarcinoma development. This model highlights a potential mechanistic link between COPD and lung cancer through alveolar stem cell exhaustion and oncogenic reprogramming.

Although this study demonstrates robust biological effects of hUC-MSC-derived secreted products, several limitations should be acknowledged. Characterization of the administered material was primarily based on vesicle morphology, particle size distribution, and the expression of a canonical exosomal marker (CD63), which together are consistent with small extracellular vesicles. However, it is increasingly recognized that MSC secretomes are heterogeneous and may comprise a mixture of membrane-bound extracellular vesicles as well as non-vesicular extracellular particles and proteinaceous assemblies [44 ‐ 46]. As such, the characterization performed in this study, while comprehensive within practical constraints, does not fully resolve the molecular complexity of the secreted material. This limitation raises the possibility that additional non-vesicular components may contribute to the observed biological effects. Accordingly, the use of the term hUC-MSC-derived secreted material may more accurately reflect the compositional diversity of the injected product. Future studies incorporating advanced fractionation strategies and multi-marker profiling will be important to further delineate the relative contributions of distinct secretome components and to refine mechanistic interpretation.

## Data Availability

All datasets generated for this study are included in the article, further inquiries can be directed to the corresponding author. The gene expression dataset can be found at the NCBI Gene Expression Omnibus [Accession number: GSE324112].
